# Data on the demographics, education, health and infrastructure: Wolaita Zone, Ethiopia

**DOI:** 10.1016/j.dib.2018.11.063

**Published:** 2018-11-15

**Authors:** Logan Cochrane, Yishak Gecho

**Affiliations:** a Carleton University, International and Global Studies, 2403R Richcraft Hall, Canada; b Institute for Policy and Development Research, Hawassa University, Hawassa, Ethiopia; c Wolaita Sodo University, Sodo, Ethiopia

**Keywords:** Ethiopia, Wolaita, Data, Demographics, Education, Agriculture, Roads, Water access

## Abstract

This data article presents a comprehensive data set about Wolaita Zone (Ethiopia), and the Woredas / Districts within it. The tables cover administrative, demographic, educational, agricultural, transport, and water aspects of the zone. The majority of the data is from 2013/2014, however, a few tables provide trend data over recent years. The evidence shows rapid population growth, significant educational challenges, limitations of health coverage, disparities of agricultural extension service provision and potable water. The data are otherwise not available to researchers and these data sets enable greater contextualization for any on-going or future research within the zone. The data were provided by the Zonal Administration in 2015, and were part of a research project that was approved by the Ethiopian Public Health Institute and supported by the Regional Health Bureau.

**Specification table**

**Table:** 

Subject area	*Demography, Health Sciences, Education, Agriculture*
More specific subject area	*Broad set of administration data from Wolaita Zone*
Type of data	*Tables*
How data was acquired	*Data were obtained from the Zonal Administration of Wolaita Zone, Southern Nations, Nationalities and Peoples’ Region, Ethiopia.*
Data format	*Analyzed*
Experimental factors	*Data used in this article were obtained from the Zonal Administration of Wolaita Zone, Southern Nations, Nationalities and Peoples’ Region, Ethiopia and is otherwise not publicly available.*
Experimental features	*Tables are employed.*
Data source location	*Ethiopia*
Data accessibility	*The data are with this article.*

**Value of the data**

• Provides comprehensive administrative data on Wolaita Zone that is not otherwise available.
• In addition to recent figures, some indictors also provide trend data, allowing researchers to assess changes over time.
• Can support on-going and future studies in Wolaita Zone, Ethiopia

## 1 Data

**Fig. 1:**
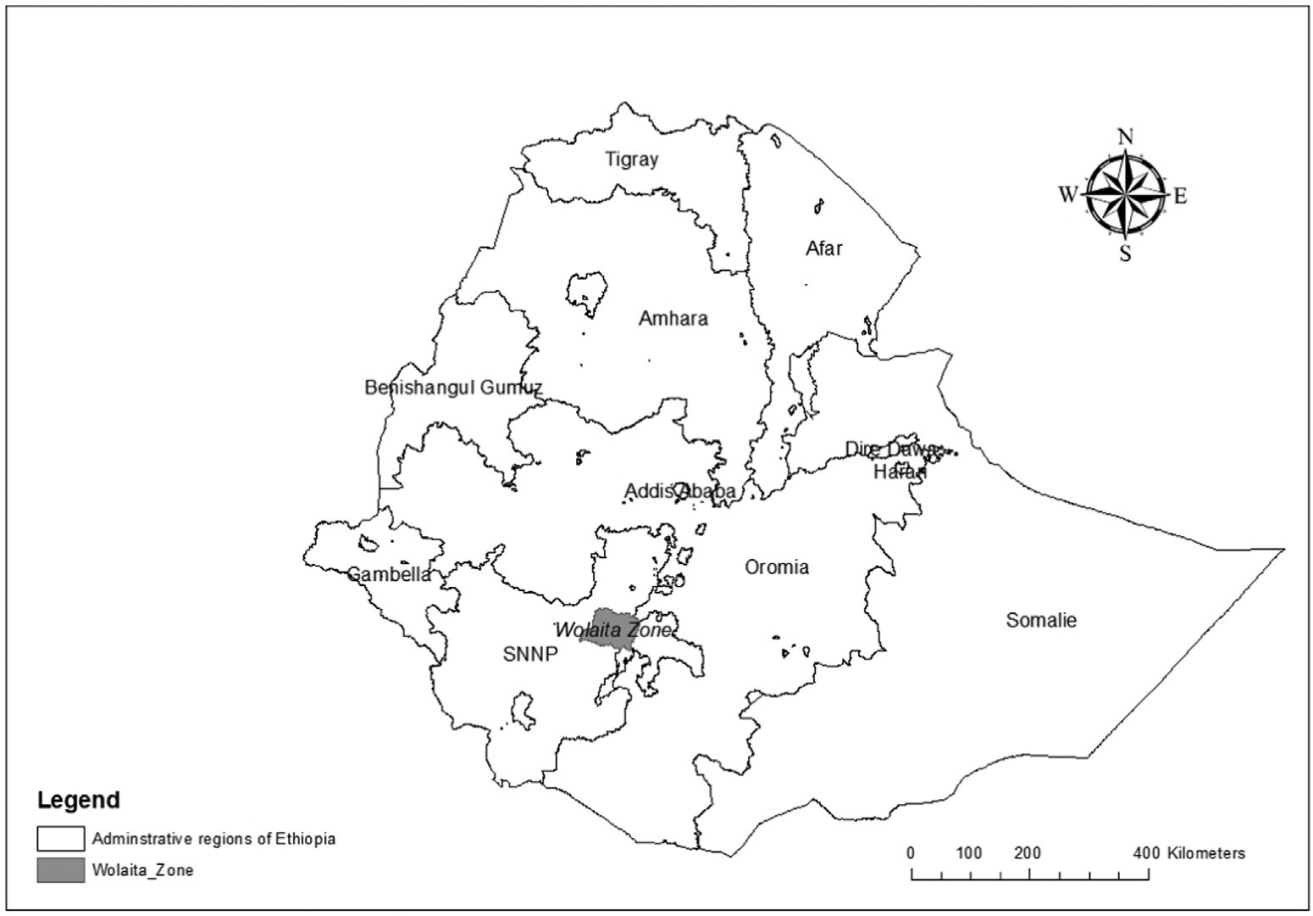
Wolaita Zone, Southern Nations, Nationalities and Peoples Regional State, Ethiopia.

The tables present a detailed picture of Wolaita Zone (Fig. 1), and the Woredas / Districts within it. The data in Table 1 outlines the Woredas/Districts and numbers of Kebeles/Communities within them, as well as their urban or rural status. The population of the zone and the woredas are presented in Table 2, providing a nine-year-period highlight significant population growth. The type, distribution, enrolment, and student-teacher ratios of schools in the zone and woredas are identified in Table 3, Table 4, Table 5. While significant progress has been made in providing educational services in Wolaita in recent decades [1], the data suggest serious challenges remain. Health institutions and service coverage are presented in Table 6, showing that higher-tier health services are unavailable to the vast majority. Table 7 outlines agricultural extension worker coverage. The road network and road types are presented in Table 8, while Table 9 outlines potable water access. Throughout, the data outline significant disparities within the zone. While the quality of data provided by statistics agencies in Africa have been called into question [2], few alternative sources exist. Recognizing the limitations, and potential politicization, of data, the objective of sharing this comprehensive administrative dataset for Wolaita Zone, and the Woredas/Districts within it, is to increase the accessibility of the government data. At present, this data are only available at the Zonal Administration office, and often obtaining the data from that can be challenging.

**Table 1:** Administrative characteristics (2006 Ethiopian, 2013–2014 Gregorian).

	**Woreda/Town**	**Capital**	**Number of Kebeles**		
			**Rural**	**Urban**	**Total**
1	Boloso Sore	Areka	25	4	29
2	Damote Gale	Boditi	31	3	34
3	Damote Woide	Bedessa	23	2	25
4	Humbo	Tebela	39	2	41
5	Sodo Zuria	Sodo	31	5	36
6	Kindo Koysha	Bale	23	2	25
7	Ofa	Gesuba	21	2	23
8	Boloso Bombe	Bombe	18	3	21
9	Damote Sore	Guguno	18	3	21
10	Kindo Didaye	Halale	19	3	22
11	Damote Pulasa	Shanto	22	1	23
12	Diguna Fango	Bitana	26	6	32
13	Sodo Town	Soddo	0	11	11
14	Areka Town	Areka	0	4	4
15	Boditi Town	Boditi	0	5	5
	**Zone Total**		**296**	**56**	**352**

**Table 2:** Demographics (1999–2007 Ethiopian, 2006/07–2014/15 Gregorian).

	**Woreda/Town**	**1999**	**2000**	**2001**	**2002**	**2003**	**2004**	**2005**	**2006**	**2007**
1	Boloso Sore	166,565	171,228	176,023	180,952	186,019	145,742	196,581	202,001	207,657
2	Damot Gale	126,946	130,500	134,155	137,911	141,772	145,742	149,823	154,018	158,330
3	Damot Woyide	91,602	94,273	97,024	99,857	102,775	105,780	108,876	112,065	115,350
4	Humbo	125,441	129,078	132,823	136,680	140,651	144,739	148,950	153,286	157,752
5	Sodo Zuriya	162,691	167,246	171,929	176,743	181,692	186,779	192,009	197,386	202,912
6	Kindo Koyisha	104,564	107,623	110,775	114,022	117,366	120,811	124,361	128,017	131,785
7	Ofa	103,870	106,887	109,993	113,192	116,487	119,879	123,373	126,971	130,677
8	Boloso Bombe	87,956	90,439	92,994	95,621	98,323	101,102	103,959	106,898	109,921
9	Damot Sore	100,683	103,625	106,654	109,775	112,990	116,301	119,713	123,227	126,847
10	Kindo Didaye	97,566	100,326	103,165	106,085	109,089	112,178	115,355	118,622	121,983
11	Damot Pulasa	105,157	108,208	111,350	114,585	117,917	121,347	124,880	128,519	132,266
12	Deguna Fanigo	96,480	99,250	102,100	105,033	108,053	111,161	114,359	117,393	120,769
13	Sodo Town	76,050	79,700	83,526	87,535	91,737	96,140	100,755	105,591	110,660
14	Areka Town	31,408	32,916	34,496	36,151	37,887	39,705	41,611	43,608	45,702
15	Boditi Town	24,133	25,291	26,505	27,778	29,111	30,508	31,973	33,507	35,116
	**Wolaita Zone**	**1,501,112**	**1,546,593**	**1,593,514**	**1,641,922**	**1,691,867**	**1,743,401**	**1,796,578**	**1,851,111**	**1,907,727**

**Table 3:** Distribution of schools (2006 Ethiopian/2013–2014 Gregorian)^a^.

	**Woreda/ Town**	**Grade**				
		**1–4**	**5–8**	**1–8**	**9–10**	**11–12**
1	Boloso Sore	3	1	38	2	0
2	Damote Gale	2	0	34	1	0
3	Damote Woyide	1	0	30	2	1
4	Humbo	7	1	43	5	1
5	Sodo Zuria	1	0	39	3	0
6	Kindo Koyisha	6	2	35	1	1
7	Ofa	6	0	24	3	1
8	Boloso Bombe	6	1	17	1	0
9	Damote Sore	4	0	20	1	1
10	Kindo Didaye	5	0	36	3	1
11	Damote Pulasa	1	0	23	1	0
12	Diguna Fango	9	0	29	3	1
13	Sodo Town	5	1	18	5	3
14	Areka Town	2	0	6	1	1
15	Boditi Town	2	0	6	1	1
**Zonal Total**		**60**	**5**	**398**	**33**	**12**

a Note: The Zonal Admiration is replicated as provided, however we wish to highlight some data points that suggest the data may not be complete. For example, five of the Woredas listed as having no 11–12 school, do have 1 (Boloso Sore, Damote Gale, Soddo Zuria, Boloso Bombe, and Damote Pulasa). This may be due to older data not be updated.

**Table 4:** Primary and secondary school enrolment by cycle (2006 Ethiopian / 2013–2014 Gregorian).

	**Woreda / Town**	**Enrollment Grade 1–4**			**Enrollment Grade 5–8**			**Enrollment Grade 9–10**			**Enrollment Grade 11–12**		
		**M**	**F**	**T**	**M**	**F**	**T**	**M**	**F**	**T**	**M**	**F**	**T**
1	Boloso Sore	18,704	17,079	35,783	8,710	7,844	16,554	1,272	920	2,192	–	–	–
2	Damot Gale	13,083	12,009	25,092	6,058	5,533	11,591	863	712	1,575	–	–	–
3	Damot Woyide	8,271	7,264	15,535	4,839	4,237	9,036	1,607	1,524	3,131	485	419	904
4	Humbo	10,987	9,892	20,879	6,824	6,027	12,851	2,564	2,012	4,576	517	405	922
5	Sodo Zuria	14,666	13,102	27,768	8,428	7,489	15,917	1,172	1,054	2,226	–	–	–
6	Kindo Koyisha	10,534	9,326	19,860	5,316	5,133	10,449	1,155	1,069	2,224	367	409	776
7	Ofa	10,585	9,638	20,223	4,408	4,316	8,724	1,047	1,155	2,202	342	337	679
8	Boloso Bombe	8,999	8,243	17,242	5,826	4,166	9,992	1,076	771	1,847	126	141	267
9	Damot Sore	7,905	7,216	15,121	3,917	3,640	7,557	1,214	1,104	2,318	408	331	739
10	Kindo Didaye	10,322	8,484	18,806	4,393	4,482	8,875	1,150	1,034	2,184	382	333	715
11	Damot Pulasa	11,536	10,346	21,882	5,384	4,768	10,152	1,241	985	2,226	–	–	–
12	Duguna Fango	9,006	8,227	17,233	4,694	4,606	9,300	1,402	1,380	2,782	79	94	173
13	Sodo Town	3,411	3,980	7,391	3,010	3,267	6,277	2,810	2,720	5,530	1,664	1,551	3,215
14	Areka Town	2,372	2,607	4,979	1,921	1,892	3,813	2,260	1,832	4,092	1,023	900	1,923
15	Boditi Town	1,305	1,627	2,932	1,164	1,338	2,502	2,094	2,188	4,282	1,261	1,006	2,267
**Zonal Total**		**141,686**	**129,040**	**270,726**	**74,892**	**68,738**	**143,590**	**22,927**	**20,460**	**43,387**	**6,654**	**5,926**	**12,580**

**Table 5:** Primary and secondary school student-teacher ratio (2006 Ethiopian/2013–2014 Gregorian).

	**Woreda/Town**	**Grade 1–4**			**Grade 5–8**			**Grade 9–10**			**Grade 11–12**		
		**Student**	**Teacher**	**Ratio**	**Student**	**Teacher**	**Ratio**	**Student**	**Teacher**	**Ratio**	**Student**	**Teacher**	**Ratio**
1	Boloso Sore	35,783	556	1:100	16,554	334	1:48	2,192	57	1:35	0	0	0
2	Damot Gale	25,092	277	1:90	11,591	391	1:30	1,575	60	1:27	0	0	0
3	Damot Woyide	15,535	191	1:82	9,076	218	1:42	3,131	84	1:38	2,092	33	1:64
4	Humbo	20,879	280	1:75	13,222	274	1:36	4,576	130	1:36	922	39	1:24
5	Sodo Zuria	27,768	327	1:84	15,917	357	1:45	2,226	70	1:32	0	0	0
6	Kindo Koyisha	21,006	389	1:54	11,181	207	1:54	2,261	55	1:41	776	19	1:41
7	Ofa	20,223	240	1:84	8,724	255	1:34	2,202	51	1:43	679	47	1:14
8	Boloso Bombe	17,742	228	1:75	9,992	307	1:32	1,858	47	1:39	267	23	1:11
9	Damot Sore	15,121	132	1:114	7,557	273	1:28	2,318	71	1:33	739	24	1:31
10	Kindo Didaye	19,207	303	1:62	8,217	143	1:57	2,165	40	1:54	699	13	1:53
11	Damot Pulasa	20,886	231	1:90	10,312	279	1:37	2,224	62	1:36	0	0	0
12	Duguna Fango	17,233	235	1:73	9,300	274	1:45	2,782	127	1:22	173	6	1:28
13	Sodo Town	7,394	230	1:32	6,277	231	1:27	5,530	183	1:30	3,215	80	1:40
14	Areka Town	4,979	90	1:55	3,813	97	1:39	4,092	95	1:43	1,923	40	1:48
15	Boditi Town	4,199	65	1:65	2,910	100	1:29	3,541	127	1:28	1,799	66	1:27
	**Zonal Total**	**273,047**	**3,774**	**1**:**77**	**14,4643**	**3,740**	**1**:**40**	**42,673**	**1,259**	**1**:**35**	**13,284**	**390**	**1**:**34**

**Table 6:** Distribution of health institutions and service coverage (2006 Ethiopian/2013–2014 Gregorian)*.

	**Woreda/Town**	**Population**	**Hospital**		**Growing health center**		**Health center**		**Health post**	
			**#**	**Ratio**	**#**	**Ratio**	**#**	**Ratio**	**#**	**Ratio**
1	Boloso Sore	202,001	0		1	1:22,454	8	1:25,261	34	1:5,944
2	Damot Gale	154,016	0		0		7	1:22,003	31	1:4,968
3	Damot Woyide	112,064	0		0		4	1:28,016	25	1:4,002
4	Humbo	153,286	0		0		7	1:25,548	39	1:3,930
5	Sodo Zuria	197,386	0		0		7	1:28,198	41	1:4,14
6	Kindo Koyisha	128,017	0		0		5	1:25,603	28	1:4,572
7	Ofa	126,971	0		0		4	1:31,742	27	1:4,702
1	Boloso Bombe	106,898	0		0		4	1:26,724	22	1:4,859
9	Damot Sore	123,228	0		0		5	1:24,646	20	1:6,161
10	Kindo Didaye	118,622	0		0		4	1:29,655	23	1:5,157
11	Damot Pulasa	128,519	0		0		5	1:25,704	23	1:5,588
12	Duguna Fango	117,651	0		1	1:70,590	6	1:70,590	32	1:3,677
13	Sodo Town	105,591	2		0		3	1:35,197	3	1:14,536
14	Areka Town	43,608	0		0		1	1:43608	0	0
15	Boditi Town	33,507	0		0		1	1:33,570	0	0

**Table 7:** Agricultural extension worker-household ratio (2006 Ethiopian/2013–2014 Gregorian).

	**Woreda/Town**	**Household size**	**Extension workers**	**Ratio**
1	Boloso Sore	40,025	101	1:396
2	Damot Gale	38,310	110	1:348
3	Damot Woyide	23,435	68	1:345
4	Humbo	32,698	148	1:221
5	Sodo Zuria	40,974	215	1:191
6	Kindo Koyisha	28,547	86	1:332
7	Ofa	27,885	83	1:336
8	Boloso Bombe	22,309	62	1:360
9	Damot Sore	25,300	76	1:331
10	Kindo Didaye	27,468	65	1:423
11	Damot Pulasa	24,706	79	1:313
12	Duguna Fango	24,061	82	1:293
13	Sodo Town	1,265	10	1:127
14	Areka Town	1,500	4	1:375
15	Boditi Town	1,363	6	1:227
	**Zonal Total**	**359,846**	**1,195**	1:**4,427**

**Table 8:** Road type and network (2006 Ethiopian/2013–2014 Gregorian).

	**Woreda/Town**	**All weather road (km)**			**Gravel weather road (km)**		**Total length in km**
		**Asphalt**	**Federal**	**Regional**	**Earth**	**Earth track**	
1	Boloso Sore	20	20	0	441	146	627
2	Damot Gale	27	0	3	14	12.5	53.5
3	Damot Woyide	3	16	11	596	115	711
4	Humbo	27.8	27.8	0	595	37	659.8
5	Sodo Zuria	36.2	62.7	38.1	135.5	49.2	321.6
6	Kindo Koyisha	0	51	54	105	81.8	186.8
7	Ofa	0	0	0	171	122	293
8	Boloso Bombe	0	0	20	143	118	281
9	Damot Sore	0	0	21.1	73	5.8	99.9
10	Kindo Didaye	0	0	26	32	32	192
11	Damot Pulasa	0	0	94.8	575	607	1,276.8
12	Duguna Fango	0	34	30	25	30	39
13	Sodo Town	27	0	0	10	9.9	38
14	Areka Town	8	8	0	0	0	16
15	Boditi Town	7.8	0	0	54	0	61.8
	**Zonal Total**	**156.8**	**219.5**	**298.0**	**2,969.5**	**1,366.2**	**4,857.2**

**Table 9:** Potable water access and coverage, Urban (U) and Rural (R)(2001–2007 Ethiopian, 2008/09–2014/15 Gregorian)^a^.

	**Woreda/Town**	**Coverage by year (%)**													
		**2001**		**2002**		**2003**		**2004**		**2005**		**2006**		**2007**	
		**U**	**R**	**U**	**R**	**U**	**R**	**U**	**R**	**U**	**R**	**U**	**R**	**U**	**R**
1	Boloso Sore	–	51	–	52	–	53	–	54	–	57	–	64	–	67
2	Damot Gale	–	35	–	37	–	40	–	43	–	48	–	62	–	98
3	Damot Woyide	29	21	30	23	45	26	49	38	70	39	86	38	–	0
4	Humbo	–	*0*	17	*0*	25	*0*	37	*0*	45	*0*	98	0	–	95
5	Sodo Zuria	37	37	42	42	57	57	69	69	78	78	86	86	92	92
6	Kindo Koyisha	5	34	10	36	16	37	56	48	64	77	74	85	–	95
7	Ofa	–	36	46	37	52	56	64	60	64	70	64	76	–	98
8	Boloso Bombe	–	0	–	0	20	20	35	35	45	45	75	75	98	98
9	Damot Sore	28	21	35	30	46	43	47	49	60	52	72	59	74	96
10	Kindo Didaye	25	28	28	29	35	30	35	54	46	61	46	61	45	69
11	Damote Pulasa	88	19	90	26	100	38	100	47	100	68	100	69	0	98
12	Duguna Fango	–	12	–	17	0	24	–	38	–	55	–	75	95	100
13	Sodo Town	34	–	38	–	40	–	52	–	65	–	77	–	77	–
14	Areka Town	0	–	34	–	39	–	52	–	64	–	76	–	91	–
15	Boditi Town	37	–	40	–	50–75	–	59	–	60	–	60	–	80	–

a Note: The missing data is not due to a lack of data, rather that these Woredas/Towns do not have urban populations. Similarly the rural population for the towns. This does not reflect the data presented in Table 1 on the presence of urban kebeles. We provide the data based on the information held at the City Administration for Wolaita Zone. One reason for the difference may be different entities (departments) collecting data and different definitions utilized.

## 2 Experimental design, materials and methods

The administrative, demographic, educational, agricultural, transport and water coverage data were obtained from the Wolaita Zone Administration of the Southern Nations, Nationalities and Peoples’ Region of Ethiopia. The tables presented here are reproduced as provided, with only minor changes to ensure consistency (e.g., on decimal point usage, spelling and formatting). For the sake of clarity, all data tables use Ethiopian terms for administrative categories: Woreda (District), Kebele (Community). With some minor corrections, the spelling utilized was that provided by the Zonal Administration. In many cases the data calculations appear incorrect (e.g., calculating ratios), but these have been left as provided by the Zonal Administration in case other unknown factors were taken into account in preparing these datasets. The evidence shows rapid population growth, significant educational challenges, limitations of health coverage, disparities of agricultural extension service provision and potable water. The data are otherwise not available to researchers and these datasets enable greater contextualization for any on-going or future research within the zone.
